# Partial isolated tear of the popliteus tendon following an in-car traffic accident: A rare cause of knee pain: A CARE-compliant case report

**DOI:** 10.1097/MD.0000000000035270

**Published:** 2023-09-15

**Authors:** Gyu-Sik Choi, Min Cheol Chang

**Affiliations:** a Cheokbareun Rehabilitation Clinic, Pohang-si, Gyeonsangbuk-do, Republic of Korea; b Department of Rehabilitation Medicine, College of Medicine, Yeungnam University, Daegu, Republic of Korea.

**Keywords:** diagnosis, magnetic resonance imaging, popliteus tendon, tear, traffic accident

## Abstract

**Rationale:**

Lesions caused by traffic accidents are often microscopic and minimal; therefore, their diagnosis can be easily overlooked. Moreover, when it is a rarely reported type of lesion, it can be even more easily undiagnosed. Isolated popliteal injuries are uncommon and have rarely been reported.

**Patient concerns:**

In this case study, we describe a right partially isolated popliteus tendon rupture that was undiagnosed for 2 years in a patient with posterior knee pain after an in-car traffic accident. A 49-year-old female patient presented with right knee pain that had persisted for 2 years and was initiated after an in-car traffic accident. The patient’s right knee pain aggravated while standing and walking. Six months after the accident, her pain was confined to the posterolateral aspect of the knee and subsequently spread throughout her right knee. The pain score was 4 on a numerical rating scale. Physical examination revealed tenderness in the posterolateral corner of the right knee. Additionally, right knee pain was reported in the terminal range of knee flexion during the passive range of motion test. Radiographs of the right knee showed normal findings.

**Diagnoses:**

A T2-weighted proton density sagittal and coronal knee magnetic resonance imaging revealed a partial-thickness tear with intrasubstance ganglion cysts at the musculotendinous junction of the popliteus tendon. No other abnormalities were observed in the patient.

**Intervention:**

Conservative treatment involved strengthening exercises and functional rehabilitation programs.

**Outcomes:**

Six months later, the knee pain almost completely subsided.

**Lessons:**

Musculoskeletal injuries caused by traffic accidents are frequently overlooked. Therefore, a detailed examination should be conducted for an accurate diagnosis. Clinicians should consider the possibility of popliteal tendon injuries in patients with posterior knee pain.

## 1. Introduction

With an increase in the number of individuals using automobiles in modern society, the number of traffic accidents has also increased. Musculoskeletal pain develops after a traffic accident and frequently persists even after the acute stage of injury. Several therapeutic methods have been applied to control post-traffic accident symptoms.^[1–3]^ Traffic accidents exert a significant impact on the body within a short period of time.^[2,3]^ In several cases, the patients do not know exactly which body areas are affected or injured during an accident.^[4–6]^ In addition, lesions caused by traffic accidents are often microscopic and of minimal size. Therefore, in several cases, despite patients complaining of persistent pain in a certain area, diagnostic studies cannot detect the pathology following a traffic accident.^[7]^ Furthermore, patients frequently exaggerate their symptoms for financial rewards from insurance companies.^[8]^ Owing to these characteristics, clinicians often do not actively conduct examinations to determine the anatomical origin of patients pain and overlook the diagnosis of obvious damage to body structures.^[9]^

Herein, we report a case of isolated partial rupture of the popliteus tendon in a patient with posterior knee pain after a traffic accident which was undiagnosed for 2 years.

## 2. Case report

A 49-year-old female patient visited our pain clinic at a university hospital complaining of right knee pain that had persisted for 2 years. Her pain began following an in-car traffic accident: while driving a sedan, she collided head-on with another sedan that crossed the centerline. In the local clinic, owing to the severe lower back, posterior neck, and left elbow pain caused by a sprain on the lumbar and cervical spines and the left elbow joint, her treatment was focused on controlling pain in the lower back, posterior neck, and left elbow. Although the patient had mentioned right knee pain, no abnormalities were detected on the knee radiographs. Subsequently, the clinician did not conduct any diagnostic studies and prescribed oral medications to control pain (celecoxib 200 mg and acetaminophen/tramadol 650/75 mg per day). Furthermore, she had no history of knee injury or pain.

**Figure 1.**
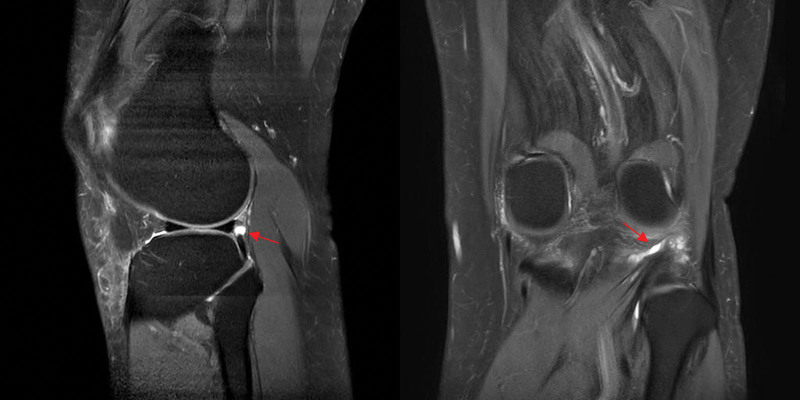
On T2-weighted proton density (A) sagittal and (B) coronal knee magnetic resonance images taken two years after the in-car traffic accident, a partial-thickness tear with intrasubstance ganglion cysts (arrows) is revealed in the musculotendinous junction of the popliteus tendon.

Due to uncontrolled knee pain, even after taking oral medications, the patient visited a pain clinic in our university hospital. The right knee pain of the patient aggravated while standing and walking. Initially, her pain was confined to the posterolateral aspect of the knee, and approximately 6 months later, it had spread all over her right knee. The degree of pain was assigned a score of 4 on a numeric rating scale (0 = no pain and 10 = worst pain imaginable). Physical examination revealed considerable tenderness at the posterolateral corner of the right knee. During the passive and active range of motion tests, right knee pain in the terminal range of knee flexion was reported. The right knee was stable in the varus and valgus stress tests during knee extension and at varying degrees of knee flexion. Additionally, the McMurray, Lachman, and anterior and posterior drawer tests were negative. The radiographs of the knees were normal. Magnetic resonance imaging (MRI) of the knee was performed. On T2-weighted proton density sagittal and coronal knee magnetic resonance imaging, partial-thickness tears with intrasubstance ganglion cysts were observed at the musculotendinous junction of the popliteus tendon (Fig. 1). No other abnormalities were observed in the patient. The patient refused arthroscopy and surgical treatment. Therefore, a strengthening exercise and functional rehabilitation program was initiated, which was conducted 3 sessions a week (40 minutes per session). At the 3 month-follow up after the initiation of the exercise and rehabilitation program, she reported that the degree of pain had reduced to NRS 2. Six months later, the knee pain almost completely subsided.

The study was approved by the local Institutional Review Board of The University Hospital. Written informed consent was obtained from the patient for publication of this case report and accompanying images.

## 3. Discussion

In this report, we present a case of partially isolated tear of the popliteus tendon after an in-car traffic accident. A knee MRI taken 2 years after the accident revealed this pathology.

In our patient, the diagnosis of popliteal tendon tear was delayed. A delayed diagnosis can result in less favorable long-term outcomes, increased operative risks, and uncontrolled pain and suffering until the injury is diagnosed.^[9]^ Missed diagnoses occur frequently, especially in patients who have experienced traumatic accidents.^[9]^ Diagnoses are frequently missed because of unconducted imaging studies and inaccurate image interpretation.^[9]^ Thus, to avoid overlooking a diagnosis in patients with traffic accidents, in the case of persistent severe musculoskeletal pain, imaging studies should be conducted actively, and clinicians should carefully interpret the images.

The popliteus is a triangular muscle that originates from the lateral surface of the lateral condyle of the femur.^[10]^ It passes downward and medially and inserts into the posterior surface of the tibia above the soleus muscle. It assists in the flexion of the knee joint, especially at the beginning of the act of bending the knee joint.^[10]^ When the knee joint is flexed, the popliteus rotates the tibia internally.^[11]^ It also prevents excessive posterior tibial translation and rotation.^[11]^ Rupture of the popliteus tendon results in posterolateral instability of the knee.^[12]^ The majority of popliteus tendon ruptures involve injury to other knee structures, such as the lateral meniscus and anterior cruciate ligament.^[12]^

Isolated rupture of the popliteus tendon is rare and is caused by a twisting injury.^[13]^ The mechanism of isolated rupture of the popliteus tendon is typically traumatic. It is caused by acute tibial external rotation in a partially flexed knee.^[14,15]^ The area posterior to the lateral tibial plateau is vulnerable to strain injury because its proximal area can be secured by the arcuate popliteal ligament.^[16]^ Most previously reported cases involved complete rupture of the isolated popliteus tendon.^[12,14,15,17–19]^ Only 1 case report has been published citing an isolated partial rupture of the popliteus tendon during sports activity (soccer).^[13]^

The diagnosis of an isolated popliteal injury based on the patient’s symptoms and physical examination is challenging because the symptoms are nonspecific, and its diagnosis requires a specialized physical examination.^[20]^ Our patient experienced posterior knee pain in the acute stage after the accident, and tenderness was observed around the area where the popliteus tendon was torn. However, posterior knee pain and tenderness can also be observed in several other knee pathologies, such as strain or myofascial pain syndrome in muscles (gastrocnemius and soleus) of the posterior knee, meniscus tears, and ligament injuries.^[21]^ Imaging studies are indispensable for the diagnosis of popliteus tendon tears.^[12,14,15,17–19]^ In almost all previous studies, MRI has been used to diagnose popliteus tendon ruptures.

In our patient, ganglionic cysts were observed at the tear site of the musculotendinous junctional area. Generally, ganglionic cysts develop in the joints or tendon sheaths. However, occasionally, a ganglion cyst originates from the muscle or intra-tendon, without being connected to the tendon sheath or adjacent joint capsule.^[22]^ Although the exact mechanism is unknown, some previous studies have proposed that ganglionic cysts may arise from myxoid degeneration of connective tissue that develops from defects of the tendon sheath or joint capsule.^[23]^ Similarly, we believe that the pieces of torn muscle and tendon tissues turned into myxoid fluid and formed a ganglion cyst in the musculotendinous junction in the space created after the tear of the popliteus muscle and tendon.

Six months after the accident, the patient’s pain was confined to the posterolateral knee area and subsequently spread throughout the knee area. The function of the injured popliteus was compensated for by other structures around the knee, and pain over the entire knee area developed from overloading on those structures.

Surgical reconstruction is indicated for the treatment of popliteus tendon tears when a popliteus tendon injury is combined with a meniscus tear, ligament rupture, or avulsion.^[12]^ However, when the popliteus tendon is isolated, knee instability rarely occurs, and the long-term risk of knee arthritis does not increase.^[12]^ Therefore, isolated popliteus tendon ruptures can be successfully treated with conservative treatment (early weight bearing, strengthening exercises, and functional rehabilitation).^[12]^

In the case of a traffic accident in South Korea, if an MRI is performed and no abnormalities are found, insurance companies often refuse to pay. Therefore, clinicians are reluctant to perform an MRI if the patient’s symptoms are not significant. This may have been the cause of the delayed diagnosis in our patient. We believe that institutional support is required to address this issue.

## 4. Conclusion

We present a case of a patient with a partially isolated tear of the popliteus tendon following an in-car traffic accident. However, the patient’s diagnosis was delayed by 2 years. A detailed examination for an accurate diagnosis should be conducted because injuries after a traffic accident are frequently missed. Clinicians should consider the possibility of popliteal tendon injuries in patients with posterior knee pain.

## Author contributions

**Conceptualization:** Gyu-Sik Choi, Min Cheol Chang.

**Data curation:** Gyu-Sik Choi, Min Cheol Chang.

**Investigation:** Gyu-Sik Choi, Min Cheol Chang.

**Methodology:** Gyu-Sik Choi, Min Cheol Chang.

**Resources:** Min Cheol Chang.

**Supervision:** Min Cheol Chang.

**Validation:** Gyu-Sik Choi, Min Cheol Chang.

**Visualization:** Gyu-Sik Choi, Min Cheol Chang.

**Writing – original draft:** Gyu-Sik Choi, Min Cheol Chang.

**Writing – review & editing:** Gyu-Sik Choi, Min Cheol Chang.
